# Xanthoma of the Jaw Bones: Cases Series and Review of Literature

**DOI:** 10.1007/s12105-024-01615-8

**Published:** 2024-03-19

**Authors:** Shankar Venkat, Mohammed N. Islam, Indraneel Bhattacharyya, Donald M. Cohen, F. James Kratochvil, Tina R. Woods, Seema Ganatra, Saja A. Alramadhan

**Affiliations:** 1 Department of Oral and Maxillofacial Pathology, University of Florida College of Dentistry, Gainesville, FL USA; 2 Department of Pathology and Radiology, Oregon Health and Science University School of Dentistry, Portland, OR USA; 3 The Medical University of South Carolina, Charleston, SC USA; 4 University of Alberta, Edmonton Clinic Health Academy, Edmonton, AB Canada; 5 University of Mississippi Medical Center, 2500 N. State St, Jackson, MS 39216-4505 USA

**Keywords:** Xanthoma, Osseous Lesion, Hyperlipidemia, Radiolucency, Multilocular

## Abstract

**Background:**

Intraosseous xanthomas are rare benign lesions sometimes associated with excess lipid production. Xanthoma of the jaw bones (XJB) was first reported in 1964, and fewer than 50 cases have been reported in the English literature to date. The etiopathogenesis of XJB is highly suggestive of a reactive process or a metabolic condition.

**Method:**

Seven cases of XJBs were retrieved from the archives of 4 oral and maxillofacial pathology services. Clinical, radiographic and histopathologic features of all these cases were retrospectively analyzed. Immunohistochemical (IHC) stains for S100 and CD68 were performed.

**Results:**

All seven cases involved the mandible. Patients’ age ranged between 13 and 69 years with an evenly distributed female to male ratio. One patient had a medical history of hyperlipidemia, but the medical and dental histories of the others were unremarkable. For most cases, XJB was an incidental finding discovered during routine radiographic examination. Swelling and cortical expansion were noted in a few cases. Radiographically, cases typically presented as either well-defined multilocular or unilocular lesions, which were either radiolucent or mixed radiolucent/radiopaque. All the lesions were treated with surgical curettage and no recurrence was observed during subsequent follow-ups. Each of the seven cases exhibited sheets of foamy macrophages. The diagnosis is established by exclusion of entities with overlapping microscopic features and involved correlation with the clinical, histological, radiographic and IHC profiles. Immunohistochemically, all the cases expressed diffuse positivity for CD68 and were negative for S100.

**Conclusion:**

XJB is a rare lesion of unknown etiology, which may mimic other benign or reactive jaw lesions. Due to its rarity and the potential diagnostic challenges it presents, clinicians must remain vigilant and consider CXJ in their differential when assessing radiolucent jaw anomalies.

## Introduction

Xanthomas are considered to be non-neoplastic reactive lesions formed by aggregates of lipid-containing histiocytes. While they can be seen in systemic conditions related to lipid metabolism disorders, they are also observed in non-systemic situations [1]. They regularly present as a mass in soft tissue and rarely present intraosseously [1, 2]. Intrabony xanthomas are often characterized by osteolysis, with loss of cortical bone. They are classified into primary and secondary bone xanthomas. Primary bone xanthomas are not associated with systemic conditions, in contrast to secondary ones [2–5].

Xanthoma of the jaw bones (XJB) was first described by Rudy & Scheingold in 1964 [6]. To date, only less than 50 cases have been reported in the English literature. The pathogenesis of this condition is unclear. XJBs are typically slow-growing lesions found in the mandible, often detected during routine radiographic examinations. However, they have the potential to cause gradual expansion, leading to extraoral swelling and significant bone destruction. Despite bone loss, the prognosis of XJB is very good. Lesions tend to regress after local curettage.

Herein, we describe 7 cases of XJB in the mandible and provide a comprehensive review of the clinicopathological features of cases reported in the literature.

## Materials and Methods

### Case Series

In the present study, cases of xanthomas of the jaw bones (XJBs) were retrieved from the archives of four oral and maxillofacial pathology services: University of Florida, University of Mississippi Medical Center, Oregon Health and Science University and University of Alberta. Patients demographics, medical history, clinical presentation, anatomical location, radiographs, treatment performed, recurrence, and follow-up period were obtained from clinical records and evaluated. All cases were microscopically reviewed, including examination of the immunohistochemical staining for S100 and CD68, to ensure the accuracy of the diagnosis.

### Literature Review

We performed a search of the English language literature between 1964 and 2020 using PubMed, Scopus, and Web of Science. The search was performed using the keyword “xanthoma of jaw bones, gnathic xanthoma, intraosseous xanthoma of the jaw, central xanthoma of the jaw” Inclusion criteria comprised case reports, case series, or retrospective studies of patients with XJB diagnosis, with sufficient clinical and histopathological data for XJB diagnosis. The following information was included from each selected study (when available): the study’s publication year, the number of cases, patients’ sociodemographic details, clinical presentation, lesion duration, treatment, recurrence, follow-up duration, and outcomes. Additionally, details regarding radiographical, histopathological, and immunohistochemical characteristics were also included.

## Results

### Case Series

Seven cases were included, all involved the mandible. The age of the patients ranged from 13 to 69 years, with an average age of 33.14 years, and a slight male predilection (*n* = 5/7). Of these, one patient had a documented medical history of hyperlipidemia (case 5). The remaining six patients’ medical and dental backgrounds were unremarkable. Clinically, in the majority of cases, the lesion was discovered during routine radiographic evaluations. However, a subset of patients exhibited swelling, with cortical expansion being a notable feature (*n* = 2/7).

**Fig. 1:**
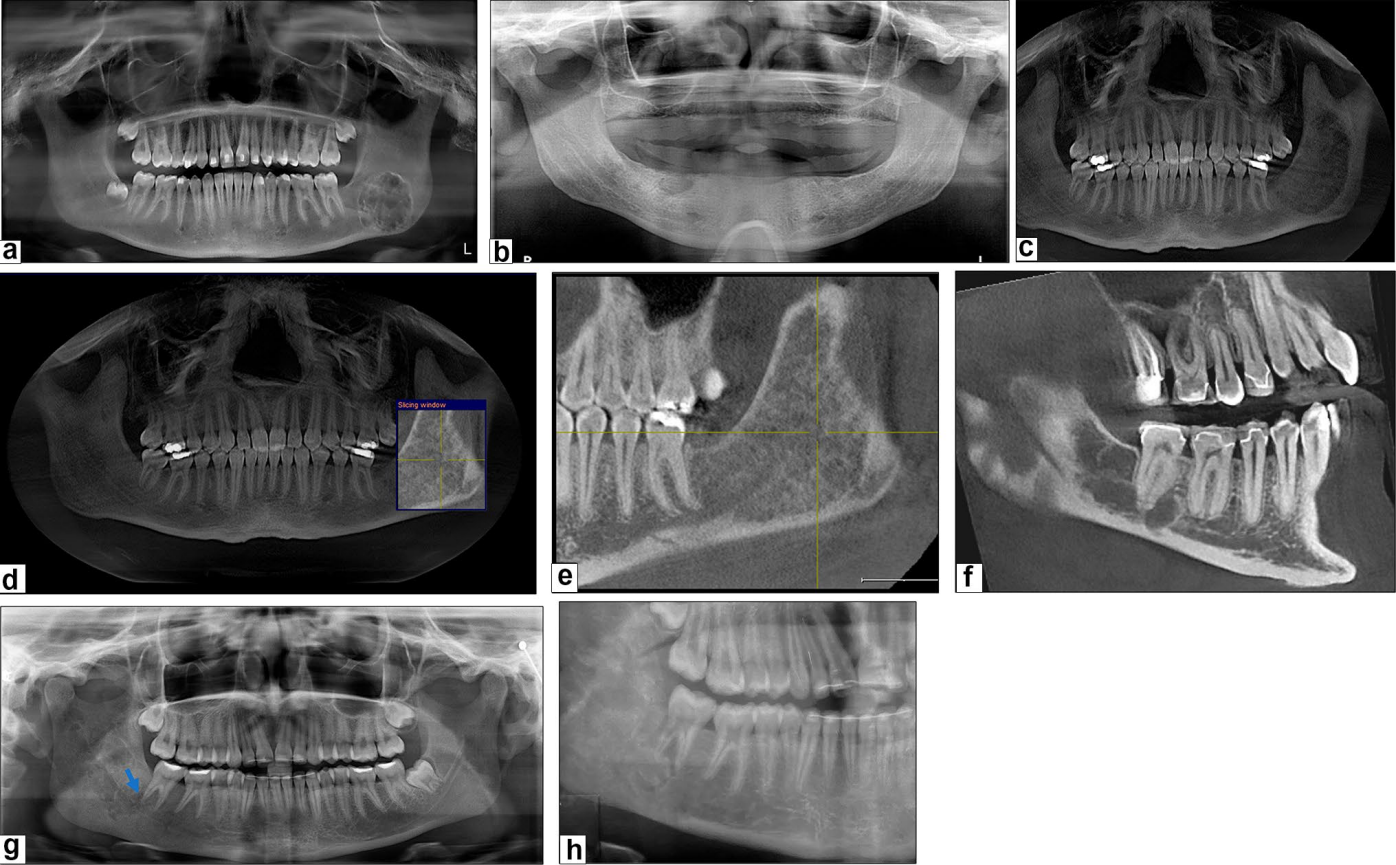
**Case 2: a** constructed panoramic radiograph shows a well circumscribed mixed radiolucent/radiopaque lesion with sclerotic border. **Case 4: b** panoramic radiograph demonstrates a unilocular radiolucent lesion at edentulous area corresponding to the site of teeth #28–30. **Case 7 c–e: c**, constructed panoramic radiograph reveals a well-circumscribed lesion with a ground-glass appearance, involving the left posterior mandible and extending to the ramus. **d, e**, post-operative follow-up radiographs show evidence of bone regeneration. **Case 3: f**, CBCT (Sagittal section) showing multilocular radiolucent lesion. **Case 1 g–h: g**, panoramic radiograph reveals an ill-defined mixed radiolucent/radiopaque lesion with evidence of distal root resorption of tooth #31 (*arrow*). **h**, post-operative follow-up radiograph showing a residual lesion that appears as an ill-defined radiolucency, with no increase in size or radiodensity

Radiographically, the presentations of xanthoma of the jaw bones (XJB) were variable. XJBs can present as either a well-defined unilocular (*n* = 5/7) (Fig. 1a–c) or multilocular (case 3) (*n* = 1/7) lesion (Fig. 1f). These can be purely radiolucent (*n* = 4/7) (Fig. 1b, c) or mixed radiolucent/radiopaque (*n* = 3/7) (Fig. 1-a). Ill-defined radiolucent (case 1) (Fig. 1g), ground glass appearance (case 7) (Fig. 1c) and root resorption (case 1) (Fig. 1g) were also reported. Bilateral involvement was observed in one hyperlipidemic patient (case 5). All the patients in our study underwent incisional biopsy, excisional biopsy, or surgical curettage. The follow-up period ranged from 6 months to 3.5 years post-surgery. During these follow-up periods, no recurrence of the lesions was observed, indicating effective and lasting treatment outcomes (Fig. 1d, e and h).

**Fig. 2:**
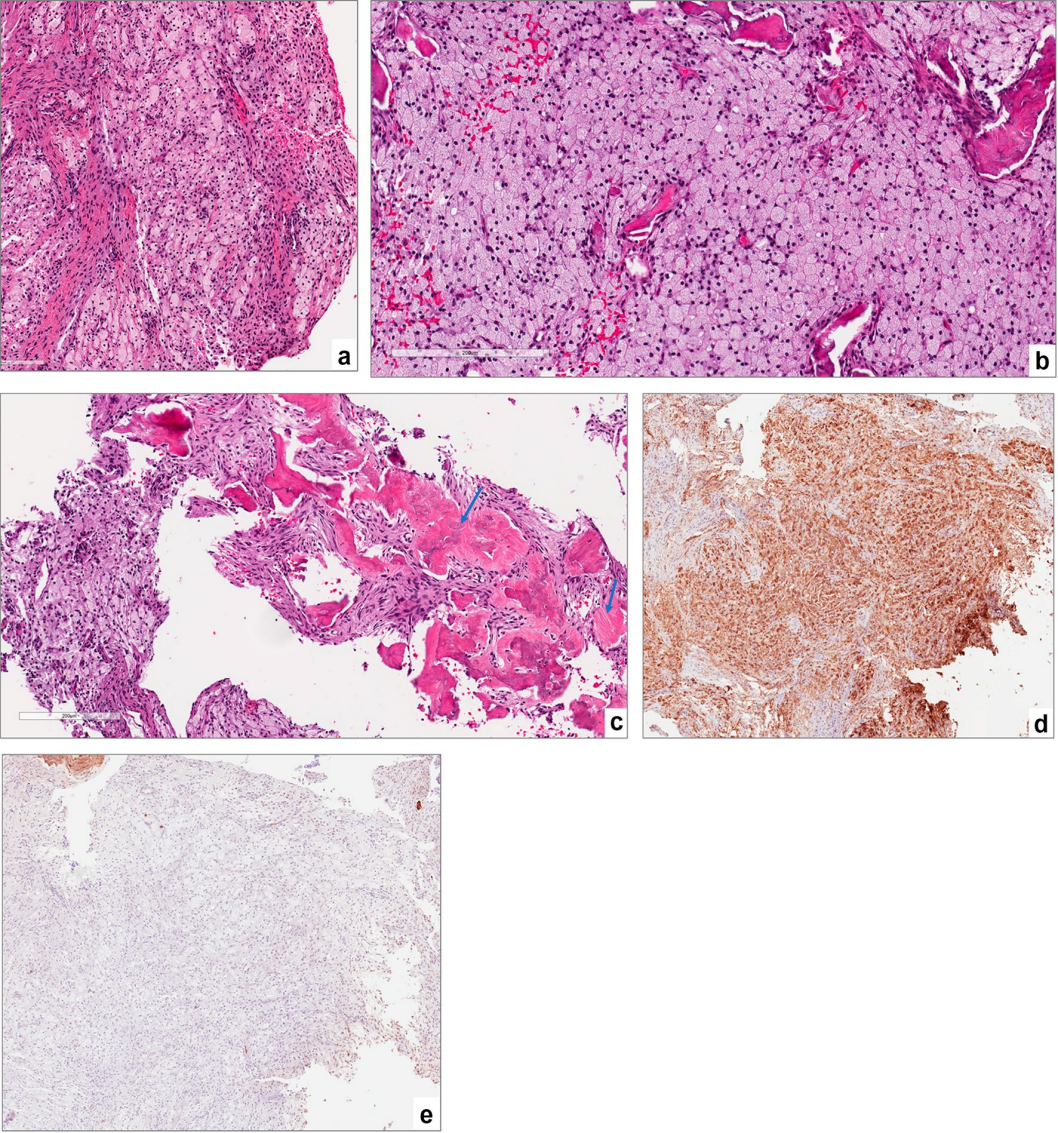
**a** sheets of foamy macrophages exhibiting granular cytoplasm with prominent nuclei and minimal intervening stroma, magnification X10 (H&E*). **b** a higher magnification of the foamy macrophages “xanthoma cells”, magnification X20 (H&E*). **c** reactive bone formation within the lesional tissue (*arrow*), magnification X10 (H&E*). **d** strong cytoplasmic positivity for CD68, magnification X4 (IHC**). **e** lesional cells lack S-100 expression, magnification X4 (IHC**). *Hematoxylin-eosin staining. ** Immunohistochemical staining

Microscopically, the typical features noted included sheets of foamy macrophages exhibiting granular cytoplasm with prominent nuclei and no significant inflammation (Fig. 2a–c). All cases demonstrated strong positive cytoplasmic staining for CD68 in the granular xanthoma cells and negative for S100 staining (Fig. 2d and e).

### Literature Review

The search returned 18 manuscripts published between 1964 and 2020, describing 46 cases of XJB.

The demographic, clinical, and radiographic features of these cases, including the current ones, are summarized in Table 1.

**Table 1:** – Demographic and Clinical Characteristics of Xanthoma of The Jaw Bones Reported in English Literature (includes current cases)

Case #	Author	Report case #	Age	Gender	Site	Clinical manifestation	Symptoms	Follow-up	Systemic conditions	Radiological findings	Immunohistochemical staining
1	Rudy & Scheingold 1964 [6]	1	49	F	Mandible	Painless swelling	Asymptomatic	NED*	Diabetes	Multilocular, radiolucent, expansion; perforation	
2	Mosby et al. 1983 [21]	1			Mandible		Asymptomatic	NA**			
3	White et al. 1986 [22]	1	29	F	Mandible		Asymptomatic	NED*	Asthma	Unilocular radiolucent; expansion of the cortical plates with partial destruction of the lingual plate	
4	Harsanyi et al. 1988 [2]	1	16	F	Mandible	Tooth displacement	Asymptomatic	Progression		Multilocular, radiolucent, expansion; root resorption	
5		2	23	M	Mandible		Asymptomatic	Progression		Unilocular, well-defined, sclerotic, mixed	
6		3	27	F	Mandible	Occlusion change	Asymptomatic	Progression		Multilocular, expansion	
7		4	13	F	Mandible	Swelling	Asymptomatic	Remained stable		Diffuse, ground glass appearance, expansion	
8		5	12	F	Mandible	Swelling	Asymptomatic	Progression		Multilocular, mixed, expansion	
9		6	15	F	Mandible		Asymptomatic	NED*		Ground glass appearance	
10		7	72	F	Mandible	Bony mass	Asymptomatic	NED*		Radiopaque, expansion	
11	Slootweg et al. 1993 [16]	1	49	M	Mandible	Swelling	Asymptomatic	NA**		Ill-defined, radiopaque, expansion	
12	Mateo et al. 2004 [4]	1	11	M	Mandible	Swelling	Asymptomatic	NED*		Multilocular, ill-defined, radiolucent, expansion	
13	Ramos-Perez et al. 2011 [23]	1	25	M	Mandible		Asymptomatic	NED*		Unilocular, ill-defined, radiolucent	+++ CD68
14	de Araujo et al. 2015 [24]	1	14	F	Mandible		Asymptomatic	NA**		Unilocular, non-corticated, radiolucent	+++ CD68, Vimentin + CD3 - CD1a, CD34, CD20, CD117, Ki-67, S-100, SMA, AE1/AE3, Desmin, HMB-45, Mycobacterium
15	Daley et al. 2015 [11]	1	56	M	Mandible	Swelling	Numbness	NED*		Unilocular, well-defined, radiolucent, expansion	+++ CD68, HLA-DR + S-100, CD1a - CD34
16		2	24	M	Mandible		Asymptomatic	NED*		Corticated, radiolucent	+++ CD68, HLA-DR + S-100 -CD34, CD1a
17		3	47	M	Mandible		Asymptomatic	NED*		Radiolucent	+++ CD68, HLA-DR + S-100 - CD34, CD1a
18		4	48	M	Maxilla		Asymptomatic	NA**		Radiolucent, expansion; perforation	+++ CD68, HLA-DR + S-100 - CD34, CD1a
19		5	22	M	Mandible		Asymptomatic	NED*		Radiolucent	+++ CD68, HLA-DR + CD1a - CD34, S-100
20	Morel et al. 2016 [15]	1	40	F	Mandible	Swelling	Pain	NED*		Multilocular, mixed, expansion	+++ CD68 - S-100, CD1a
21	Rawal et al. 2017 [13]	1	22	F	Mandible		Asymptomatic	NED*		Unilocular, well-defined, punched-out, radiolucent	All cases were +++ for either CD68 or CD163
22		2	25	M	Mandible		Asymptomatic	NED*		Multilocular, well-defined, sclerotic, radiolucent	
23		3	15	M	Mandible		Asymptomatic	NED*		Unilocular, well-defined, radiolucent	
24		4	12	F	Mandible		Asymptomatic	NED*		Unilocular, well-defined, sclerotic, radiolucent	
25		5	58	F	Maxilla	Swelling	Pain	NED*		Unilocular, well-defined, non-corticated, radiolucent	
26		6	49	F	Mandible		Asymptomatic	NED*		Unilocular, well-defined, punched-out, radiolucent	
27		7	36	M	Mandible		Asymptomatic	NED*		Unilocular, well-defined, punched-out, radiolucent, erosion, root resorption	
28		8	35	M	Mandible		Asymptomatic	NED*		Unilocular, well-defined, punched-out, radiolucent	
29		9	53	F	Maxilla	Tooth mobility	Pain	NED*		Unilocular, well-defined, non-corticated, radiolucent, expansion, root displacement; root resorption	
30		10	63	F	Mandible		Asymptomatic	NED*		Unilocular, well-defined, punched-out, radiolucent, root displacement; root resorption	
31	Brooks et al. 2018 [8]	1	16	M	Mandible	Swelling	Asymptomatic	NED*	Hyperlipidemia / Vitamin D deficiency	Multilocular, ill-defined, mixed, expansion; erosion, root displacement	+++ CD68 + S-100 (0.3%) - CD1a
32	Olson et al. 2018 [9]	1	15	M	Mandible	Swelling and tooth mobility	Pain	NED*	Noonan syndrome	Unilocular, well-defined, radiolucent, expansion; erosion	+++ CD68 - S-100, CD1a, langerin
33	Saha et al. 2018 [20]	1	30	M	Mandible		Asymptomatic	NED*		Ill-defined, radiolucent, erosion	
34	Cunha et al. 2018 [14]	1	35	F	Maxilla	Swelling	Asymptomatic	NED*		Unilocular, well-defined, mixed, expansion; perforation	+++ CD68 - Desmin, S-100, CD34, AE1/AE3
35	Whitehouse et al. 2018 [10]	1	22	F	Mandible	Swelling	Asymptomatic	Progression	Orofacial granulomatosis	Non-corticated, mixed, expansion, root resorption	+++ CD68, Factor 13a - S-100, CD1a
36	de Arruda et al. 2019 [3]	1	23	M	Mandible		Asymptomatic	NED*		Unilocular, well-defined, sclerotic, radiolucent	+++CD68 - S-100
37		2	21	F	Mandible		Asymptomatic	NED*		Unilocular, ill-defined, punched-out, radiolucent	+++CD68 - S-100
38		3	45	F	Mandible		Asymptomatic	NED*		Unilocular, ill-defined, punched-out, radiolucent	+++CD68 - S-100
39		4	19	F	Mandible		Asymptomatic	NED*		Multilocular, well-defined, punched-out, radiolucent	+++CD68 - S-100
40		5	13	F	Mandible		Asymptomatic	NED*		Multilocular, ill-defined, punched-out, mixed, expansion; erosion	+++CD68 - S-100
41	Wilkinson et al. 2020 [12]	1	28	F	Mandible		Asymptomatic	NA**		Multilocular, radiolucent	+++ CD68 - S-100, CD1a
42		2	17	F	Mandible		Asymptomatic	NA**		Unilocular, well-defined, radiolucent	+++ CD68, - S-100, CD1a
43		3	27	M	Mandible		Asymptomatic	NA**		Multilocular, sclerotic, radiolucent	+++ CD68 - CD1a
44		4	33	M	Mandible		Asymptomatic	NA**		Multilocular, radiolucent	**Xanthoma cells**: Positive for CD68, CD45, alpha-1-antitrypsin (AAT), IgG, IgA, Lysozyme Negative for CD1α, S100 **Spindled cells**: Negative for α-SMA, Desmin **Hyaline globules**: Positive for Lysozyme, AAT, IgG, IgA, Pas-diastase, Masson’s trichrome (Bright red)
45		5	14	F	Mandible		Asymptomatic	NA**		Multilocular, sclerotic, radiolucent	**Xanthoma cells**: Positive for CD68, CD45 Negative for CD1α, S100, HMB45 **Spindled cells**: Positive for CD99, α-SMA Negative for Desmin, Myogenin, Pancytokeratin
46		6	12	F	Mandible		Asymptomatic	NA**		Radiolucent	+++ CD68, - S-100, CD1a
47	Present cases	1	36	M	Mandible	Minimal swelling	Asymptomatic	NA**		Ill-defined radiolucent lesion, with mild cortical expansion, erosion, and root resorption	+++CD68 - S-100
48		2	13	F	Mandible		Asymptomatic	NED*		Unilocular mixed radiolucent-opaque lesion	+++CD68 - S-100
49		3	15	F	Mandible		Asymptomatic	NED*		Multilocular radiolucent lesion	+++CD68 - S-100
50		4	53	M	Mandible	Swelling	Asymptomatic	NA**		Unilocular radiolucent lesion	+++CD68 - S-100
51		5	69	M	Mandible		Asymptomatic	At one-year follow-up, lesions are stable with no increase in size nor increase in radiodensity	hyperlipidemia, hypertension and congenital blindness	Bilateral well circumscribed radiolucent lesion	+++CD68 - S-100
52		6	29	M	Mandible		Asymptomatic	NA**		Mixed radiolucent/opaque lesion	+++CD68 - S-100
53		7	17	M	Mandible		Asymptomatic	NED*		Well circumscribed, mixed radiolucent-opaque lesion with a ground-glass appearance	+++CD68 - S-100

NED* No Evidence of Disease; N/A** Not Applicable

N/A** Not Applicable

+ scarce positive cells

− negative immunoreaction

## Discussion

While soft-tissue xanthomas are relatively common lesions associated with accumulation of cholesterol-rich material in hyperlipidemic patients, its bony counterpart is rare [1, 2]. A recent advancement in our understanding of lipid protein metabolism has enabled us to determine that circulating lipoproteins increasingly deposit into adjacent tissues following local trauma or hemorrhage. The cholesterol passes between the vascular endothelial cells into different sites of the body as non-degradable sterols that are later eliminated by tissue macrophages through phagocytosis [3]. This process of elimination gives rise to “foamy cells” or xanthoma cells, a characteristic feature of xanthomas. Additionally, it has been suggested that these lesions could be manifestations of a pre-existing pathological condition, such as degenerative alterations associated with different types of lesions, including aneurysmal bone cyst, traumatic bone cavity, fibrous dysplasia, or giant cell tumors [4, 5].

The central or intraosseous xanthomas that particularly affect the appendicular and axial bones are typically linked to lipid disorders associated with endocrine or metabolic diseases [7]. A review of the current literature suggested very few patients had systemic diseases associated with intraosseous xanthomas. Brooks et al. 2017 described xanthoma of the jaw bones (XJB) in a 16-year-old male with hyperlipidemia and vitamin D deficiency [8]. Another case was reported of a 15-year-old male having XJB in conjunction with Noonan Syndrome, an autosomal dominant disorder that is associated with the development of giant cell lesions in the jaws [9]. Other studies outlined the connection of XJB with conditions such as diabetes and orofacial granulomatosis [6, 10]. The etiology of non-systemic XJB is currently unknown. A recent study reported the lack of a targeted DNA mutation in intraosseous xanthomas after investigating 50 of the most common cancer-related genes [9]. Due to lack of certainty in etiopathogenesis, intraosseous xanthomas are thought to be caused by a reparative or reactive process of a previously diagnosed pathological condition [10–12].

Previous reports have suggested that XJBs occur more commonly in males [11]. However, our comprehensive analysis showed a slight female predilection (44.4%, *n* = 20 males and 55.6%, *n* = 25 females), in contrast to the previous reports and the findings of this study. The age range of patients affected with XJB is from 11 to 72 years of age, with an average age of 30.01 years which aligns with the present study [12]. No age differences between women and men were noted. Although 90% of the cases affected the mandible, recent publications described 4 cases occurring in the maxilla. XJB in the maxilla represents only 7.4% of all the cases reported [11, 13, 14]. In 32.1% (*n* = 17) of cases, XJBs demonstrated swelling, tooth mobility or displacement [2, 4, 6, 8–11, 13–16]. Importantly, a large proportion of cases exhibited cortical erosion or perforation, and resorption of roots. In some cases, the increased bony expansion caused considerable destruction of the jaw. The associated or adjacent teeth are almost always vital with little to no tenderness on percussion. Pain was an uncommon finding, and almost 90% of the cases were detected during routine radiographic examination as in our cases. Dull pain and mucosal numbness were the most common findings in symptomatic cases [9, 11, 13, 15].

XJBs exhibit radiographic features that overlap with many common lesions of the jaw, however, cone-beam computed tomography (CBCT) imaging may offer a helpful diagnostic tool [14]. Based on the available literature, the majority of XJBs show multilocular or unilocular, punched out, radiolucent lesions with or without sclerotic borders. Although several studies, including the present study, have reported mixed and even radiopaque radiographic presentation [2, 3, 8, 10, 14, 15]. Harsanyi et al. reported seven cases of XJB, two of which exhibited a ground glass appearance [2]. This variation may be attributed to reactive bone formation (Fig. 2-c), dystrophic calcification, or the deposition of calcified material, similar to that seen in fibrous dysplasia [16].

The radiographic characteristics are not definitive for diagnosing XJB but suggest an initial differential diagnosis of lesions such as odontogenic tumors/cysts or periapical inflammation [14]. Therefore, histopathological and immunohistochemical analyses are essential for diagnosis. The dominant feature of this entity is the presence of sheets of lipid-laden histiocytes (xanthoma cells), which are also known as foamy cells, with abundant cytoplasm and small hyperchromatic nuclei. These cells are surrounded by fibrous connective tissue with no significant inflammation. Other histological findings include the presence of giant cells, lamellar and woven bone fragments and dystrophic calcifications [11, 12].

Diagnosing XJB histopathologically can be challenging, especially in secondary inflamed cases. Other intraosseous lesions, such as non-ossifying fibroma (NOF) and benign fibrous histiocytoma (BFH), also display focal collections of foamy histiocytes and multinucleated giant cells. These cells are distributed within a fibrous connective tissue stroma that often demonstrates a whorling or storiform pattern [11, 13]. Granular cell odontogenic tumor (GCOT) also falls in the list of xanthomatous lesions. Histologically, GCOTs demonstrate islands or strands of inactive odontogenic epithelium interspersed within a background of granular mesenchymal cells. The absence of both fascicular fibrous stroma and odontogenic epithelium islands aids in distinguishing XJB from other intraosseous xanthomatous lesions. Reactive foamy histocytes are also seen in periapical inflammatory lesions. Yet, these histocytes are often intermixed with varying numbers of chronic and/or acute inflammatory cells and should not be interpreted as XJB [3]. Nonetheless, clinical presentation (i.e., non-vital teeth) can aid in establishing a definitive diagnosis. Other disorders that present with xanthomatous cells include Rosai-Dorfman, Erdheim-Chester, and Gaucher diseases. Gaucher disease, the most common lysosomal storage disease, can also affect the gnathic bones [17]. In Gaucher disease, a genetic mutation leads to the accumulation of lipids in various organs, including bones. The histopathologic exam demonstrates vacuolated, lipid-laden reticuloendothelial cells (Gaucher’s cells) infiltrating the connective tissue, characterized by enlarged, granular cytoplasm and round, displaced nuclei [17]. Furthermore, systemic metabolic and lipid diseases, such as type II and III hyperlipidemia and diabetes mellitus, should be included for a complete differential diagnosis [15]. Hyperlipidemia, a condition characterized by elevated levels of lipids (fats) in the blood, is categorized into different types based on specific lipid profiles [18]. Elevated lipid levels can lead to the accumulation of fats in tissues and may cause subcutaneous and tendon xanthomas, xanthelasma (cholesterol deposits building up under the skin), and corneal arcus (deposition of lipid in the peripheral cornea). However, bony manifestations are uncommon, but can be the first sign of the disease [15, 19]. Unlike intraosseous xanthomas, the foamy macrophages associated with hyperlipidemia are associated with cholesterol crystal clefts, inflammatory reactions, and giant cells, which can result in fibrosis [15]. Since these diseases frequently show histopathological overlap, an immunohistochemical analysis as well as evaluation of pertinent medical history is important to distinguish them from XJB [15].

Immunohistochemically, xanthomas demonstrate strong cytoplasmic positivity for CD68, a marker used to identify the activated macrophages [3]. Besides CD68, supplementary markers such as S100, factor XIIIa + and CD1a are useful in separating xanthomas from other diseases. These three markers help identify neural cells, histiocytes and Langerhans cells respectively [15]. It is observed that both S100 and CD68 are diffusely positive in Rosai-Dorfman disease, Erdheim disease and Gaucher disease. Similarly, S100 and CD1a are consistently positive for Langerhans cell histiocytosis thus making them reliable in distinguishing xanthomas from Langerhans disease. Although both NOF and BFH exhibit variable positivity to CD68, they are strongly positive for factor XIIIa + making this immunohistochemical tool important in differentiating tumors with fibrohistiocytic lineage from xanthoma of bone [3, 11, 15].

XJB generally has an excellent prognosis. Though XJB can cause extensive bone destruction, curettage is the widely accepted method of treatment. This study is consistent with most reported cases, which demonstrated complete healing after surgical intervention. However, of the 46 cases reported in the literature, only 5 cases exhibited progression [2, 10]. Research indicates that curettage of XJB lesions is effective in promoting bone regeneration [3]. Interestingly, Saha et al. presented a case of XJB where initial surgical removal of the lesion was followed by a second surgical intervention to completely remove the lesion [20]. During the second procedure, newly formed woven bone with absence of xanthoma cells was noted in the central portion of the lesion, supporting adequacy of a single surgical intervention.

## Conclusion

Xanthomas of the jaw bones (XJBs) are rare, and their pathogenesis, particularly in the absence of systemic diseases, remains unclear. These lesions have a strong affinity for the mandible and affect both genders equally across a broad age range. They usually present as asymptomatic osteolytic lesions, which may mimic other benign or reactive jaw lesions. While XJBs grow slowly, they can cause significant bone destruction if left untreated over extended periods. Fortunately, the majority of the lesions exhibit excellent prognosis following simple surgical curettage. Due to their rarity and the potential diagnostic challenges they pose, clinicians should remain vigilant and consider XJB in their differential when assessing radiolucent jaw anomalies.

## Data Availability

No datasets were generated or analysed during the current study.
